# Impact of Welding Current on Weld Formation in Variable Polarity Plasma Arc Welding: A Numerical and Experimental Analysis

**DOI:** 10.3390/ma18051122

**Published:** 2025-03-01

**Authors:** Ruiqing Lang, Yongquan Han, Yonglin Ma

**Affiliations:** 1 Department of Materials Science and Engineering, Inner Mongolia University of Science and Technology, Baotou 014010, China; langruiqing2@163.com; 2 Inner Mongolia Key Laboratory of New Metal Material, Baotou 014010, China

**Keywords:** variable polarity plasma arc, arc thermal-force fluctuations, critical current difference, keyhole behavior, numerical simulation

## Abstract

The characteristics of a variable polarity plasma arc (VPPA) and the keyhole behavior significantly influence weld formation. This study investigates the impact mechanism of welding current on weld formation by examining both arc thermal-force output and keyhole behavior through a combination of numerical analysis and experimental methods. A three-dimensional transient arc model with alternating loading of electrode negative (EN) and electrode positive (EP) polarity arcs is developed based on magnetohydrodynamics and is enhanced by user-defined scalars (UDS). The analysis of the arc characteristics reveals that the arc in the EN phase exhibits a larger arc penetration force and keyhole digging effect, while a divergence of the arc occurs in the EP phase. The thermal force of the arc exhibits periodic variation with changes in arc polarity. EN and EP arcs associated with “critical current difference” have minimal thermal fluctuations, minimal fluctuations in the keyhole dimensions (the keyhole long-axis size and keyhole area fluctuation ranges are 4.5–5.2 mm and 78–83 mm^2^, respectively), and the best keyhole stability and weld bead formation. Otherwise, the fluctuation of the keyhole long-axis size and keyhole area can be very large, which may lead to an unstable keyhole molten pool and poor weld formation.

## 1. Introduction

Aluminum alloy is essential for manufacturing lightweight equipment in an energy-efficient and low-emission manner, leading to its widespread adoption as a high-performance metal across modern industries [1,2]. Variable polarity plasma arc welding (VPPAW) is an effective welding method designed for aluminum and its alloys [3]. It resolves the challenge of cathodic cleaning of the oxide layer on aluminum alloy surfaces while preventing tungsten electrode burning loss. This method facilitates single-sided welding with the double-sided formation of medium-thickness aluminum alloys, achieving high-quality welds with enhanced efficiency [4]. Consequently, VPPAW is considered a promising welding method for aluminum alloys. However, this welding method is hindered by several limitations, including complex multi-parameter matching, a narrow process range, inadequate molten pool stability, and a low process repetition rate. These factors impede both its industrial application and technological advancement.

The plasma arc provides heat and force to the workpiece. Under high constraint conditions, the variable polarity plasma arc (VPPA) rapidly alternates between electrode negative (EN) polarity and electrode positive (EP) polarity, resulting in distinct characteristics during each phase. Zhang et al. [5] introduced an innovative, flexible VPPA system to address the instability issues during the transverse welding process, highlighting the significant influence of arc characteristics on welding stability. Lang et al. [6] discovered that alterations in welding parameters can induce periodic thermo-mechanical oscillations within the keyhole molten pool. An appropriate parameter range is crucial for maintaining the stability of the keyhole molten pool. The arc plasma provides the heat and force to the keyhole molten pool, and differences in thermal-mechanical outputs from the plasma arcs may lead to instability of the sensitive keyhole molten pool, ultimately resulting in poor weld formation. Thus, precisely defining and understanding the influence of welding parameters on arc characteristics is essential as a fundamental prerequisite for ensuring molten pool stability and achieving high-quality weld formation. Although the VPPA characteristics are relatively complicated, existing research on VPPA remains limited, with most studies mainly focusing on specific application levels. Pan et al. [7] investigated the distribution of arc temperature during the EN and EP stages. The findings revealed that the arc temperature during the EP stage is lower than in the EN stage, and the arc distribution exhibits a divergent pattern. Jiang et al. [8] assessed the thermal efficiency of the VPPA and discovered that the arc efficiency during the EN phase surpassed that of the EP phase, providing a qualitative explanation for this phenomenon. The physical characteristics of high-temperature plasma pose significant challenges for experimental investigation. With the continuous advancement and application of computer technology, researchers are increasingly utilizing simulation techniques to quantitatively analyze the properties of arc plasma. Shinichi et al. [9] developed a two-dimensional alternating current arc model to investigate the thermal transfer characteristics of the arc in the EN and EP stages. The findings indicate that the total heat flux transmitted from the arc to the workpiece in the EN stage is greater than that in the EP stage. Chen et al. [10] proposed a time-sharing conductive arc model that varies with arc polarity. The radial temperature field, arc pressure, and plasma flow field distribution of a VPPA were quantitatively analyzed. However, the study did not investigate the underlying mechanism by which welding parameters influence arc stability and weld formation in VPPAW. The three-dimensional VPPA model accurately represents the spatial characteristics of the arc, and the calculation results are more consistent with reality. This model also facilitates the analysis of arc behavior across different positions and directions. In addition, previous experimental research demonstrated that for VPPAW, the welding current exerts the most substantial influence on weld formation [11,12,13,14]. Consequently, we prioritized investigating the influence mechanism of welding current on weld formation by focusing on arc stability.

The consistent presence of the keyhole throughout the K-PAW welding procedure is critical for determining weld quality [15]. Moreover, the variations in arc characteristics significantly influence the dynamic keyhole behavior. In turn, the keyhole behavior reflects the underlying changes in the arc characteristics. Therefore, the keyhole behavior serves as a critical indicator for verifying and validating the effects of arc dynamics throughout the welding process. Researchers have comprehensively explored sensing technology for monitoring keyhole penetration behavior in DC plasma arc welding. Dong et al. [16] captured signals characterizing the characteristic state of the keyhole through arc light sensors, revealing that keyhole dimensions play a critical role in determining keyhole stability, welding process stability, and weld joint quality. In addition, researchers have elucidated the keyhole penetration process through numerical analysis [17,18]. However, current studies have not sufficiently explored the intricate relationship between fluctuations in keyhole size and aspects such as weld formation and the stability of the molten pool.

In summary, a foundational understanding of the distribution of arc characteristics during VPPAW in both the EN and EP stages has been established, and preliminary insights into the changes in keyhole behavior have been obtained. Nevertheless, the impact of welding current on welding formation remains ambiguous when examined from the perspectives of arc characteristics and keyhole behavior.

In this study, we conducted a quantitative analysis of the impact mechanism of welding current on weld formation. Specifically, we employed a three-dimensional transient magnetohydrodynamic calculation model to examine the stable output of arc thermal forces and investigated the influence of welding current on keyhole behavior. The findings provide valuable insights and theoretical guidelines for achieving good weld formation.

## 2. Experimental Procedure

**Figure 1:**
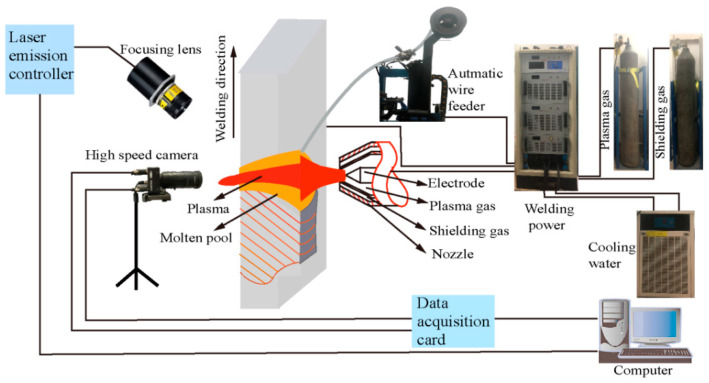
Diagram of VPPAW and the high-speed camera system.

As illustrated in Figure 1, the VPPAW platform comprises a power supply VPPA-300 (Self-developed by Beijing University of Technology, Beijing, China), a plasma arc welding torch PWM-300 (Victor Technologies Group, Inc., Clayton, MO, USA), a water-cooling mechanism (Shandong Aotai Electric Co., Ltd., Jinan, China), an automatic wire feeder (Chengdu Welding Research Technology Co., Ltd., Chengdu, China), and gas cylinders containing pure argon for both plasma and shielding gases, with a flow rate of 15 L/min. The workpiece is made of 2A14-T6 aluminum alloy, measuring 120 mm in length, 60 mm in width, and 6 mm in thickness. A tungsten electrode with a diameter of 4.8 mm and a 60° tip angle was employed. The nozzle orifice measures 4.0 mm in diameter, while the torch standoff distance is 5 mm. The welding wire used is ER2319, with a wire feeding rate maintained at 120 mm/min. Table 1 presents the chemical composition of the 2A14 aluminum alloy and the filler wire, as determined by inductively coupled plasma technology ICAP7200 (Thermo Fisher Scientific Inc., Shanghai, China), along with a comparative analysis based on references [19,20]. The duty cycle ratio for the EN to EP phases is 21 ms to 4 ms.

The welding parameters are preliminarily recommended based on previous studies [21,22]. Furthermore, a substantial current during the EP phase (*I*_ep_) is recommended to effectively remove the oxidation layer while minimizing the degradation of the tungsten electrode [23]. Consequently, the EN phase (*I*_en_) current was set to 150 A, while the EP phase (*I*_ep_) currents were set at 150 A, 170 A, 190 A, 210 A, and 230 A, respectively. The welding parameters are outlined in Table 2, with *Q* representing the plasma gas flow rate.

**Figure 2:**
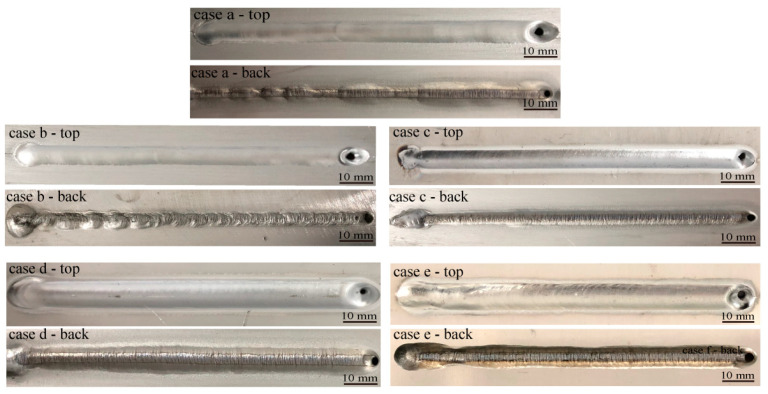
Weld formation with different welding currents.

The experimental results demonstrate that when *I*_en_ and *I*_ep_ were set to 150 A and 190 A, the weld formation is good, as is shown in case c in Figure 2. When *I*_ep_ is 150 A or 170 A, the weld formation on the top and backside is uneven. When *I*_ep_ is 210 A, the weld formation is satisfactory. However, due to the larger *I*_ep_ generating more heat, the corresponding weld width is larger than in *I*_ep_, which is 190 A. If *I*_ep_ increases to 230 A, there will be an undercutting tendency on the top of the weld during the early stage of the welding process, and the backside of the weld will exhibit a broader melting width, potentially attributed to the increased thermal energy produced by the large current.

## 3. Simulation and Experimental Results and Discussion

### 3.1. Simulation Results and Discussion

#### 3.1.1. Assumptions and Governing Equations

The generation of VPPA is a complex physical process. To enhance the manageability of the model, the following assumptions were established: (1) Plasma arc is assumed to be in a state of local thermodynamic equilibrium and local chemical equilibrium, and the parameters of plasma thermodynamics and transmission performance are only considered as a function of temperature [24]. (2) The energy dissipation process caused by fluid viscosity and heat exchange between different fluid parts is neglected. (3) The arc is a continuous medium [25] and an incompressible fluid in laminar flow [26]. (4) The reabsorption of arc radiation and the radiation loss of the entire wavelength can be ignored. (5) The VPPA is symmetric with respect to the xoz plane. Based on these assumptions, the governing equations are formulated as follows.

The continuous formula [26] is as follows:

(1) ∂ρ/∂t+div(ρν)=0

where *ρ* represents the density; *t* depicts the time; and v denotes the velocity vector.

The momentum conservation formulas are represented below [27]:

(2) $$\frac{\partial (\rho u)}{\partial t}+div(u\rho \nu )=J_{y}B_{z}-J_{z}B_{y}-\frac{\partial P}{\partial x}+\frac{\partial }{\partial x}\left\{\mu \left[2\frac{\partial u}{\partial x}-\frac{2}{3}\left(\frac{\partial u}{\partial x}+\frac{\partial v}{\partial y}+\frac{\partial w}{\partial z}\right)\right]\right\}+\frac{\partial }{\partial y}\left[\mu \left(\frac{\partial u}{\partial y}+\frac{\partial v}{\partial x}\right)\right]+\frac{\partial }{\partial z}\left[\mu \left(\frac{\partial w}{\partial x}+\frac{\partial u}{\partial z}\right)\right]$$

(3) $$\frac{\partial (\rho v)}{\partial t}+\mathrm{div}(v\rho \nu )=J_{z}B_{x}-J_{x}B_{z}-\frac{\partial P}{\partial y}+\frac{\partial }{\partial y}\left\{\mu \left[2\frac{\partial v}{\partial y}-\frac{2}{3}\left(\frac{\partial u}{\partial x}+\frac{\partial v}{\partial y}+\frac{\partial w}{\partial z}\right)\right]\right\}+\frac{\partial }{\partial z}\left[\mu \left(\frac{\partial v}{\partial z}+\frac{\partial w}{\partial y}\right)\right]+\frac{\partial }{\partial x}\left[\mu \left(\frac{\partial v}{\partial x}+\frac{\partial u}{\partial y}\right)\right]$$

(4) $$\frac{\partial (\rho w)}{\partial t}+\mathrm{div}(w\rho \nu )=J_{x}B_{y}-J_{y}B_{x}-\frac{\partial P}{\partial z}+\frac{\partial }{\partial z}\left\{\mu \left[2\frac{\partial w}{\partial z}-\frac{2}{3}\left(\frac{\partial u}{\partial x}+\frac{\partial v}{\partial y}+\frac{\partial w}{\partial z}\right)\right]\right\}+\frac{\partial }{\partial x}\left[\mu \left(\frac{\partial w}{\partial x}+\frac{\partial u}{\partial z}\right)\right]+\frac{\partial }{\partial y}\left[\mu \left(\frac{\partial w}{\partial y}+\frac{\partial v}{\partial z}\right)\right]$$

where *u*, *v*, and *w* denote the velocity components along the x, y, and z axes, respectively; *J*_x_, *J*_y_, and *J*_z_ denote the current densities corresponding to the x, y, and z axes, respectively; *B*_x_, *B*_y_, and *B*_z_ represent the magnetic flux density along the x, y, and z axes, respectively. *P* denotes pressure, while *μ* signifies viscosity.

The energy conservation formula can be represented as follows [10]:

(5) $$\frac{\partial (\rho c_{p}T)}{\partial t}+\mathrm{div}(\rho c_{p}T\nu )=\frac{\partial }{\partial x}\left(\kappa \frac{\partial T}{\partial x}\right)+\frac{\partial }{\partial y}\left(\kappa \frac{\partial T}{\partial y}\right)+\frac{\partial }{\partial z}\left(\kappa \frac{\partial T}{\partial z}\right)+\frac{{J_{x}}^{2}+{J_{y}}^{2}+{J_{z}}^{2}}{\sigma }+\frac{5\kappa_{B}}{2e}\left(J_{x}\frac{\partial T}{\partial x}+J_{y}\frac{\partial T}{\partial y}+J_{z}\frac{\partial T}{\partial z}\right)-S_{R}$$

where *T* represents the temperature, *C*_P_ denotes the specific heat capacity, *κ* stands for the thermal conductivity, *K*_B_ denotes the Boltzmann constant, and *e* represents the amount of charge of an electric charge. The second and third terms on the right side of the energy conservation formula correspond to Joule heating and heat transfer due to electron movement, respectively. *S*_R_ represents the radiation loss. The Maxwell equations are resolved to derive the Joule heating term. Maxwell equations include Ohm’s theorem, the current continuity formula, and the Poisson formula for the magnetic vector potential [10].

Ohm’s theorem is as follows:

(6) $$J_{x}=-\sigma \frac{\partial \varphi }{\partial x}=-\sigma E_{x},\;J_{y}=-\sigma \frac{\partial \varphi }{\partial y}=-\sigma E_{y},\;J_{z}=-\sigma \frac{\partial \varphi }{\partial z}=-\sigma E_{z}$$

The current continuity formula is

(7) $$\frac{\partial }{\partial x}\left(\sigma \frac{\partial \phi }{\partial x}\right)+\frac{\partial }{\partial y}\left(\sigma \frac{\partial \phi }{\partial y}\right)+\frac{\partial }{\partial z}\left(\sigma \frac{\partial \phi }{\partial z}\right)=0$$

The Poisson formula for the magnetic vector potential is as follows:

(8) $$-\nabla^{2}A_{x}=\mu_{0}J_{x},\;-\nabla^{2}A_{y}=\mu_{0}J_{y},\;-\nabla^{2}A_{z}=\mu_{0}J_{z}$$

(9) $$B_{x}=\frac{\partial A_{z}}{\partial y}-\frac{\partial A_{y}}{\partial z},\;B_{y}=\frac{\partial A_{x}}{\partial z}-\frac{\partial A_{z}}{\partial x},\;B_{z}=\frac{\partial A_{y}}{\partial x}-\frac{\partial A_{x}}{\partial y}$$

where ϕ represents the electric potential; $E_{x}$, $E_{y}$, and $E_{z}$ represent the electric field intensities alongside the x, y, and z axes, respectively; $A_{x}$, $A_{y}$, and $A_{z}$ signify magnetic vector potential alongside the x, y, and z axes, respectively; $B_{x}$, $B_{y}$, and $B_{z}$ correspond to the magnetic flux densities alongside the x, y, and z axes, respectively; and $\mu_{0}$ represents the vacuum permeability.

#### 3.1.2. Computational Domain and Boundary Conditions

**Figure 3:**
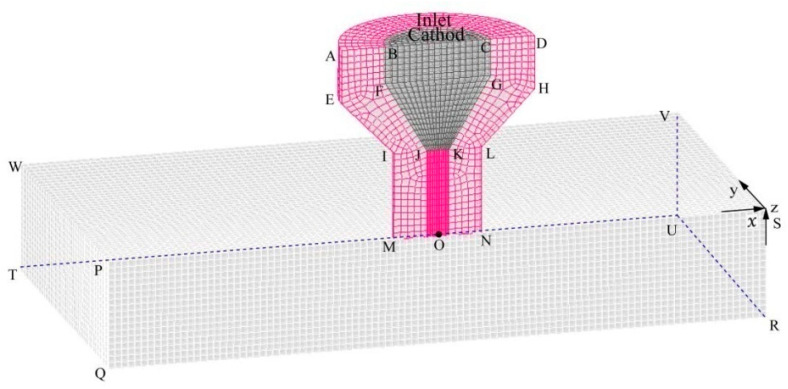
Computational domain of VPPA.

Considering the distribution characteristics of the VPPA, the structural design of the nozzle, and computational efficiency, a three-dimensional symmetric solution domain for the arc was constructed, as depicted in Figure 3. The geometric modeling encompasses the compression nozzle (red area), tungsten electrode (dark gray area), and arc column area (gray area). QRUT represents the workpiece surface (assumed to be a plane). The tungsten electrode features a diameter of 4.8 mm, with the tip precisely angled at 60 degrees. The tip is modeled as a circular surface measuring 1 mm in diameter, while the internal shrinkage of the electrode features 4 mm. The height of the arc column area measures 5 mm, and the nozzle aperture has a diameter of 4.0 mm.

A. Tungsten electrode boundary layer treatment

A low-temperature sheath layer exists between the tungsten electrode and plasma arc interface, as illustrated in the BFJKGC interface shown in Figure 3. In the sheath, significant gradients in plasma arc temperature, particle density, and voltage are observed, preventing the plasma from achieving local thermodynamic equilibrium [28]. Therefore, the boundary layer requires special treatment in the calculation process. The LTE diffusion approximation technique [29] is employed to address the boundary layer at the interface between the tungsten electrode and the plasma arc. This method requires a grid size of 0.1 mm or smaller on the electrode surface to ensure that the temperature in the grid remains at or above 7000 K. The elevated temperature in the boundary layer increases the tungsten electrode tip conductivity, maintaining the consistency of the electric field [30]. Given that the tungsten electrode remains solid, its boundary BFJKGC was designated as a wall surface. In addition, according to Park et al. [31,32], cathode and anode grid sizes in the range of 0.1–0.4 mm can achieve both a reasonable solution and eliminate the need for special treatment of the sheath region, where the two electrodes deviate from local thermodynamic equilibrium. In our study, the minimum size of the tungsten electrode surface is fixed to 0.1 mm, and the minimum grid size for the boundary layer within the arc region is determined to be 0.3 mm. The mesh discretization is performed using the finite volume method (FVM) [33].

B. Heat transfer between the tungsten electrode boundary layer and the plasma arc

According to Shinichi et al. [9], the impact of the boundary layer is addressed by incorporating an additional energy source term associated with the boundary layer at the coupling interface. The density of electrons and ions can be expressed as follows [34]:

(10) $$\begin{array}{l} j_{e}=\left\{ \begin{array}{ll} j_{R}\;\;\;\;\;\;\;\;\;\; & if\left|\vec{j}\cdot \vec{n}\right|-j_{R}>0\\ \left|\vec{j}\cdot \vec{n}\right| & if\left|\vec{j}\cdot \vec{n}\right|-j_{R}\leq 0 \end{array}\right.\\ j_{i}=\left|\vec{j}\cdot \vec{n}\right|-j_{e} \end{array}$$

where $\vec{n}$ represents the surface normal vector, $j_{e}$ denotes the electron current density, and $j_{i}$ stands for the ion current density.

Energy source terms related to the boundary layer in EN and EP phases must be included in the model periodically throughout the VPPAW process. The energy source on the tungsten electrode’s surface during EN and EP stages is as follows [9]:

(11) $$H_{c}^{\mathrm{en}}=-\left|\vec{j_{e}}\right|\varphi_{c}+\left|j_{i}\right|V_{i}-\varepsilon \alpha_{1}T^{4}\;\;\;\;\;\;\;\;\;\;\;\;\;\;\left(EN\;\;phase\right)$$

(12) $$\;\;H_{c}^{\mathrm{ep}}=\left|\vec{j_{e}}\right|\varphi_{ai}-\varepsilon \alpha_{1}T^{4}\;\;\;\;\;\;\;\;\;\;\;\;\;\;\;\left(EP\;\;phase\right)$$

The first term on the right side of Equation (11) represents the cooling effect of thermionic emission electrons on the tungsten electrode, the second term represents the thermal impact of ions on the cathode, while the third term represents the radiation heat loss of the tungsten electrode. The first term on the right of Equation (12) represents the heating effect of electrons on the tungsten electrode, and the second term represents the radiation heat loss of the tungsten electrode. $\varphi_{c}$ denotes the work released from tungsten electrode materials, and $\varphi_{\mathrm{ai}}$ represents the work released from aluminum alloy materials. $V_{i}$ represents the ionization potential of argon gas, *ε* denotes the radiation loss coefficient, and $\alpha_{1}$ is the Stephen Boltzmann constant.

C. Current boundary conditions

A current density is applied at the tip of the tungsten electrode. The process of alternating polarity involves the switching between electron emission and reception by the tungsten electrode. The tungsten electrode acts as the electron emission source during the EN phase, with the conductive regions of the tungsten electrode tip designated as sigma1. In the EP stage, the workpiece serves as the electron emission source, and the tungsten electrode begins to receive electrons. The conductive surface of the tungsten electrode tip is more extensive, denoted as sigma2. In addition, following the principle of minimum voltage in arc physics, the electron emission ability gradually weakens from the tungsten electrode tip to the surrounding area, resulting in a Gaussian distribution of the current density. Consequently, the current density at the tip of the tungsten electrode varies according to Equation (13):

(13) $$\left\{ \begin{array}{l} J_{z}=-\frac{I}{\pi r_{a}^{2}}\exp(-b(x^{2}+y^{2}))\\ \left(EN\;phase:I=I_{en},\;\;r_{a}=sigma1\right)\\ \left(EP\;phase:I=I_{ep},\;\;r_{a}=sigma2\right) \end{array}\right.$$

where *J*_z_ represents the current density of the tungsten electrode tip, *b* denotes a constant, taken as −1.94 [10], and *r*_a_ is the conductive area of the tungsten electrode tip. The preliminary experimental results of our research indicate that the arc distribution range in the EN phase is more concentrated than that in the EP phase, which is caused by the gradual cleaning of the oxide film from the center to the periphery of the arc [6]. In addition, based on the measured shape of the EP and EN arcs, the heating range of the EP arc is about 40% larger than that of the EN one [35]. Therefore, we adopt sigma2 = 1.4 sigma1, with sigma1 being 0.6 mm and sigma2 being 0.85 mm. *I*_en_ and *I*_ep_ represent the welding current in the EN and EP stages, respectively. The potential of the QRUT on the workpiece surface is set to 0. The remaining outer boundaries are set as insulated boundary conditions, with no current passing through.

D. Thermal boundary conditions.

The following section will elucidate the boundary condition setting by referencing Figure 3 in the previous text. The thermal boundary condition at the symmetry plane (AEIMONLHD and PQRS surfaces) is ∂T/∂n=0 with the adoption of a three-dimensional symmetric model. The surface temperature of the argon gas inlet is set to 1000 K, and the upper surface of the QRUT workpiece, the gas outlet on the side (PQTW, SRUV, UVWT), and the gas outlet on the upper surface (WPSV) is set to 300 K. In VPPAW, the main function of the nozzle is to fix the electrode and induce thermal-mechanical compression on the plasma arc. Therefore, the nozzle surface boundary AEIMDHLN is set as a fixed wall surface. Additionally, due to the cooling effect of the circulating water inside the nozzle, the nozzle surface boundary AEIMDHLN is set as a constant temperature boundary with a temperature of T = 1000 K [36] (the melting point of copper is 1357.77 K).

E. Momentum boundary conditions

The argon gas inlet is designated as a velocity inlet, with the inlet velocity maintained as a constant value determined by the specified plasma gas flow rate. The gas outlet on the lateral side (PQTW, SRUV, and UVWT in Figure 3) and the gas outlet on the upper surface (WPSV in Figure 3) are defined pressure outlets.

#### 3.1.3. Material Physical Properties and Numerical Implementation

The thermophysical properties of argon, including the density, electrical conductivity, thermal conductivity, and viscosity, vary with temperature [37]. The thermophysical properties of the tungsten electrode materials are given in previous studies [37,38]. The tungsten in the model exists in the solid phase, and its material properties are interpolated into the solid-phase material. The computational object is an incompressible steady fluid, and the governing equations are solved using the second-order upwind solver of the SIMPLE algorithm. First, the initial property parameters are introduced into the flow field to calculate the property parameters of all nodes in the fluid domain. Subsequently, boundary conditions are established and source terms are defined. The Maxwell equations are solved using the current property parameters to determine the electromagnetic force and Joule heat. Electromagnetic field calculation results are simultaneously incorporated into the flow field to modify the temperature distribution and material properties within the fluid domain. Finally, the updated temperature distribution and material properties are incorporated into the Maxwell equations for iterative computation until convergence is achieved.

#### 3.1.4. Results and Discussion

**Figure 4:**
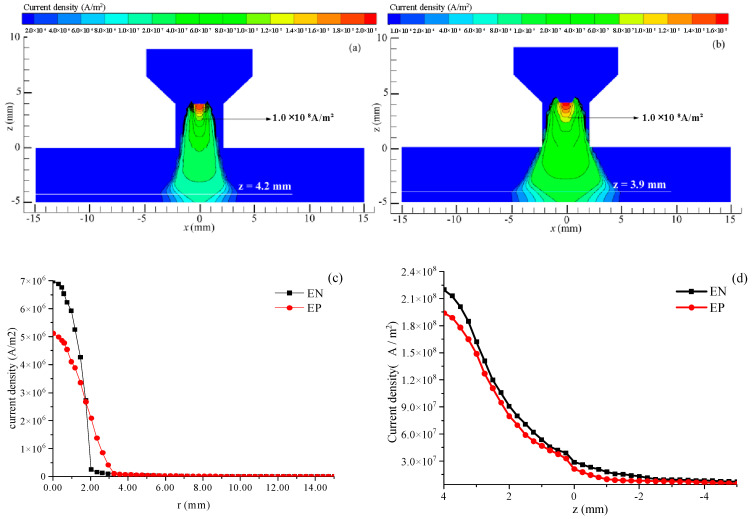
Current density distribution of VPPA. (**a**) EN phase; (**b**) EP phase; (**c**) on the workpiece surface; (**d**) along the axis direction.

The VPPA characteristics under identical current conditions (*I*_en_ = *I*_ep_ = 150 A) were investigated. Figure 4a,b depicts the current density distribution of the plasma arc during the EN and EP stages. The current density of the plasma arc exhibits a Gaussian distribution for z > −4.2 mm during the EN stage and z > −3.9 mm during the EP stage. The maximum current density is observed at the tip of the tungsten electrode, with the peak current density in the EN stage (2.0 × 10^8^ A/m^2^) surpassing that observed in the EP phase. In addition, the current density distribution range during the EP stage exhibits a broader range both inside and outside the nozzle compared to the EN stage, explaining the more divergent nature of the arc in the EP stage. As illustrated in Figure 4c, the peak current density values for the EN and EP stages are 6.9 × 10^6^ A/m^2^ and 5.1 × 10^6^ A/m^2^, respectively. Furthermore, the current density for the EN and EP stages decreases to a minimum at approximately 2.5 mm and 3.5 mm from the arc center, respectively. As illustrated in Figure 4d, the current density decreases from top to bottom along the arc axis direction, with the current density during the EN stage surpassing that of the EP stage. This observation indicates that the current density of a VPPA exhibits periodic variations corresponding to the periodic changes in arc polarity.

**Figure 5:**
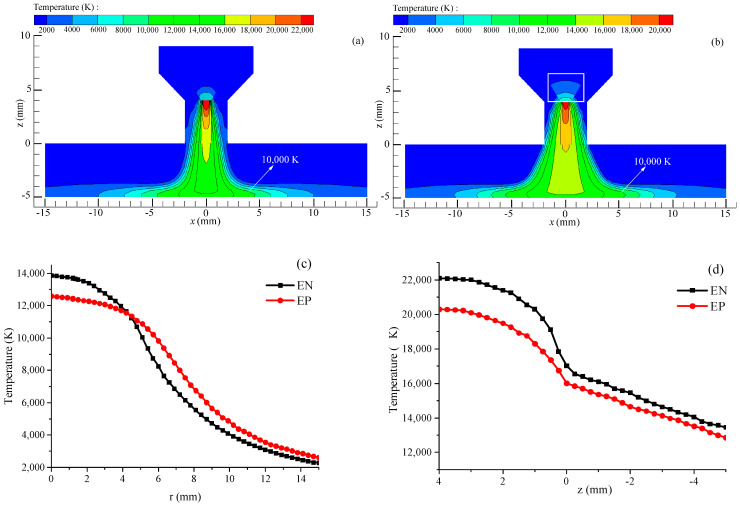
Temperature of the VPPA. (**a**) EN phase; (**b**) EP phase; (**c**) on the workpiece surface; (**d**) along the axis direction.

Figure 5 illustrates the temperature distribution of the VPPA. As shown in Figure 5a,b, the arc temperature during the EN and EP stages outside the nozzle presents a “bell-shaped” distribution, with the highest temperature appearing near the tungsten electrode tip. Notably, the maximum arc temperature during the EN stage exceeds that of the EP stage. In the EP phase, the tungsten electrode tip experiences heating over a larger surface area and at elevated temperatures (as depicted in the white box in Figure 5b), which enhances the hot electron emission area while simultaneously reducing the arc current density. When the arc transitions from the EP stage to the EN stage, the reduced temperature at the tungsten tip, along with the decreased area of the electron emission surface, results in an elevated current density at the tungsten tip. This phenomenon explains the elevated peak temperature and arc contraction in the EN stage. As observed in Figure 5c, the temperature of the arc on the workpiece gradually decreases from the center towards the surrounding regions. Within a specific radial distance range, the arc temperature during the EN stage is higher than in the EP stage. However, this trend reverses once the radial distance surpasses approximately 5 mm. As illustrated in Figure 5d, the arc temperature progressively diminishes from the tungsten electrode tip towards the workpiece surface alongside the axial direction, with the arc temperature during the EN phase exceeding that of the EP stage. This observation indicates that the temperature of a VPPA exhibits periodic variations corresponding to the periodic changes in arc polarity.

**Figure 6:**
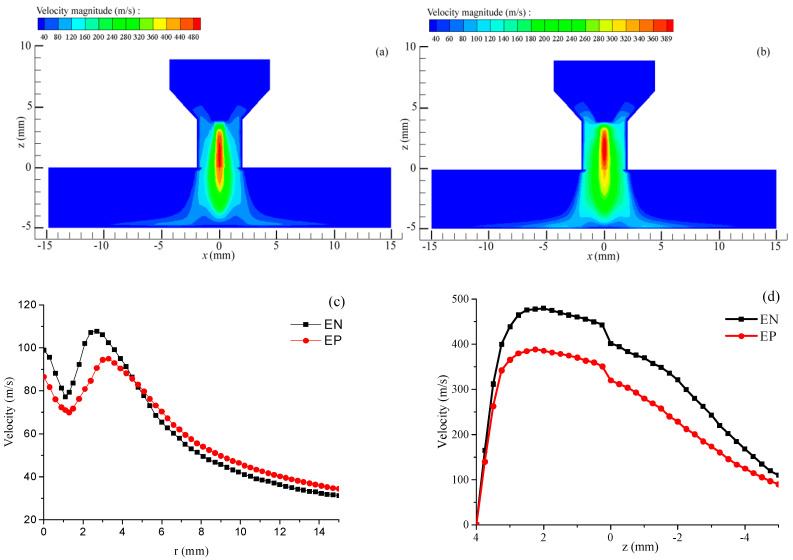
Velocity of the VPPA. (**a**) EN phase; (**b**) EP phase; (**c**) on the workpiece surface; (**d**) along the axis direction.

Figure 6a,b shows that the peak arc plasma flow velocity during the EN stage reaches 480 m/s, exceeding the maximum velocity of 389 m/s in the EP stage. The distribution range of arc plasma velocities during the EP stage is broader than that in the EN stage. Figure 6c demonstrates that the arc plasma flow velocity during both the EN and EP stages initially decreases, followed by an increase and a final decrease in the radial direction. The peak flow velocities in plasma during the EN and EP stages are recorded at 2.2 mm and 2.8 mm from the center of the workpiece surface, corresponding to 110.9 m/s and 95.5 m/s, respectively. The extensive distribution range of the arc during the EP stage results in a relatively large radial distance corresponding to the maximum flow velocity. Figure 6d depicts the velocity field along the axis of the VPPA, with z = 0 representing the nozzle end face. The plasma flow velocity increases and then decreases inside the nozzle, with the highest value appearing inside the nozzle. During the EN stage, the maximum plasma velocity is 480 m/s at a vertical distance of 2.5 mm beneath the tungsten electrode’s tip. In contrast, the highest plasma flow velocity in the EP phase is 389 m/s, observed at a vertical distance of 1.9 mm under the tungsten electrode’s tip. The peak arc plasma flow velocity during the EN phase surpasses that of the EP phase, occurring closer to the workpiece surface. This indicates a more pronounced jet effect of the arc plasma during the EN phase. Figure 6d indicates that the axial plasma flow velocity during the EN phase surpasses that observed in the EP stage. This analysis supports the conclusion that the jet effect of the VPPA experiences periodic fluctuations corresponding to the cyclical changes in arc polarity.

**Figure 7:**
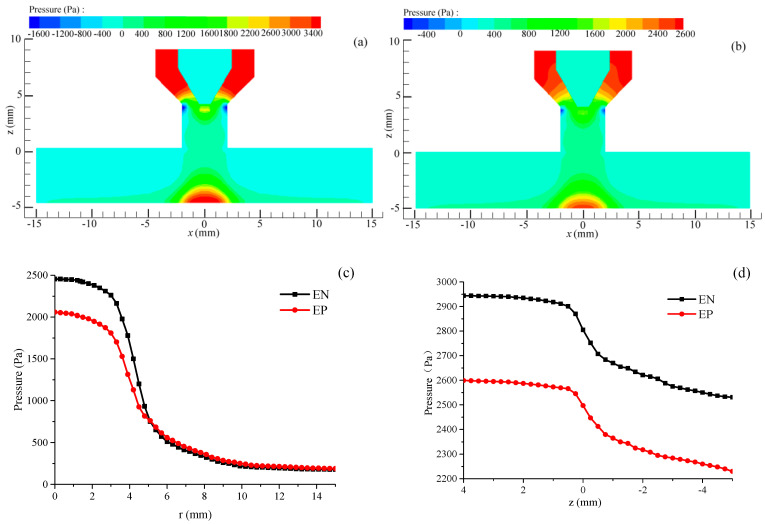
The changes in arc pressure: (**a**) EN phase, (**b**) EP phase, (**c**) on the workpiece surface, and (**d**) along the axis direction.

Figure 7a,b illustrates that the arc pressure at the center of the workpiece reaches its peak, with the maximum pressure during the EN stage surpassing that of the EP stage. Furthermore, the arc pressure exerted on the workpiece decreases radially in both the EN and EP stages. Figure 7c illustrates that the decrease rate in arc pressure during the EN stage is more significant compared to the EP stage as the radial distance increases. When the radial distance is below 5 mm, the arc pressure during the EN stage exceeds that during the EP stage. However, once the radial distance surpasses 5 mm, the arc pressure values reverse, suggesting that the arc is predominantly focused in the EN stage. As illustrated in Figure 7d, the arc pressure gradually decreases from the tip to the workpiece along the central axis of the arc. Notably, whether within or outside the nozzle, the arc pressure during the EN stage consistently exceeds that of the EP stage by approximately 350 Pa (*I*_en_ = *I*_ep_ = 150 A). This analysis suggests that the excavation effect produced by the VVPA on the workpiece experiences periodic fluctuations corresponding to the cyclical changes in arc polarity.

#### 3.1.5. Experimental Verification

**Figure 8:**
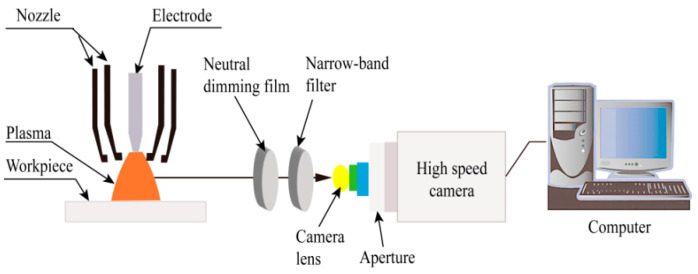
High-speed camera arc image acquisition system.

A high-speed camera arc image acquisition system (Figure 8) was developed to measure the temperature of the VPPA using the argon standard temperature technique. This system employs a narrowband filter with a central wavelength of 794.8 nm and a bandwidth of 3 nm ± 1.5 nm. The ArI 794.8 nm arc image without overexposure was captured by adjusting the optical path. The high-speed camera’s acquisition frame frequency is set to 1000 frames per second. In order to minimize equipment instability and the influence of noise on the arc image created by the surrounding environment, preprocessing techniques such as noise reduction (filtering), interpolation, and symmetry enhancement should be applied to the arc image.

**Figure 9:**
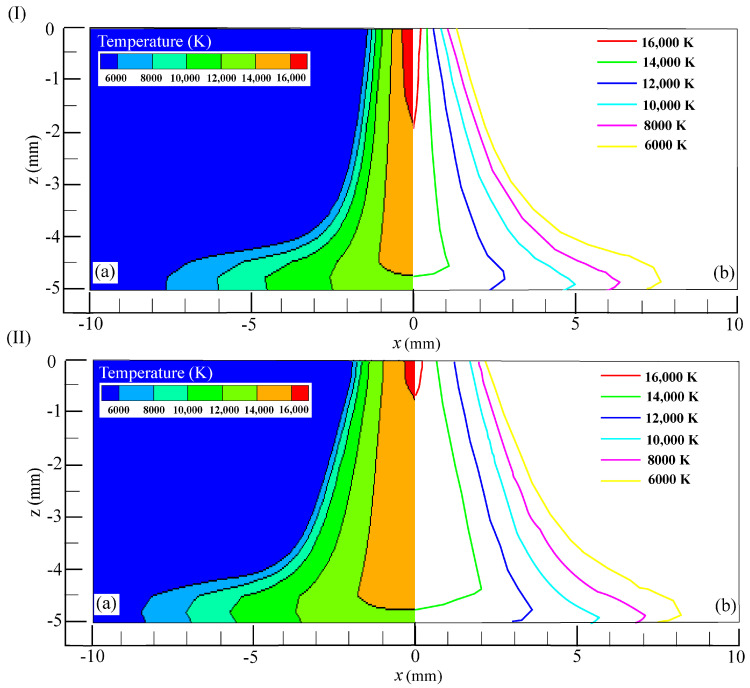
Comparison of calculated and measured temperatures of VPPA: (**I**) EN phase ((**a**) simulation result; (**b**) experimental result) and (**II**) EP phase ((**a**) simulation result; (**b**) experimental result).

Figure 9I(a,b) depicts the arc temperature distributions obtained via simulation and experimental measurement in the EN stage, respectively. The comparison results indicate that the radial distribution of the measured isotherm exhibits greater divergence compared to the simulated ones, particularly within the range of 2–4 mm from the arc central axis. This discrepancy may be attributed to deviations in the theoretical calculations of the given tungsten electrode conductivity and the measurement error. Subsequently, the arc temperature distributions derived from both simulation and experimental measurements in the EN and EP stages are analyzed for comparative evaluation. Generally, the computed arc temperature distribution agrees well with the experimental data. For illustrative purposes, an example of the isotherm distribution at 10,000 K is provided. When z is −3 mm in the EN stage, the calculated radial distance corresponding to the isotherm is approximately 1.7 mm and the measured radial distance is about 2.0 mm. For the EP stage, the calculated radial distance for the isotherm is approximately 1.8 mm and the measured radial distance corresponding to the isotherm is about 2.2 mm. In summary, the arc temperature distribution in the EN and EP stages agrees well with the experimental measurements, thereby validating the accuracy of the numerical model.

#### 3.1.6. Influence Mechanism of Welding Current on VPPA Characteristics

**Figure 10:**
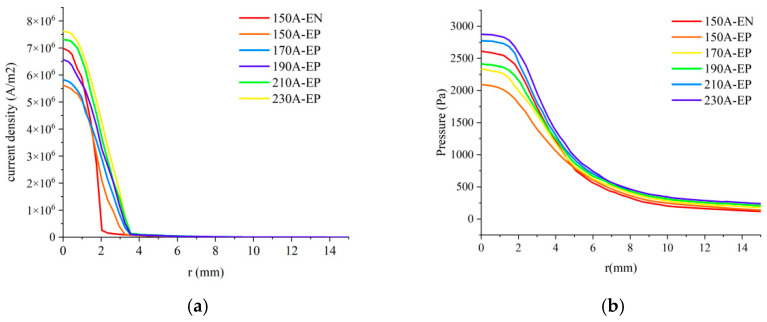
Influence of increased current during the EP phase on the characteristics of VPPA (**a**) current density; (**b**) pressure.

Figure 10 depicts the characteristics of VPPA with a current of 150 A during the EN phase (*I*_en_) and varying currents of 150 A, 170 A, 190 A, 210 A, and 230 A during the EP phase (*I*_ep_). Figure 10a,b depicts the radial variation of current density and arc pressure of VPPA on the workpiece surface at the z = −5 mm cross-section. It is evident that peak values of the arc current density and arc pressure in both the EN and EP stages are located at the central axis of the arc. During the EP stage, both the current density and arc pressure increase as the current level rises. When *I*_en_ is 150 A and *I*_ep_ remains below 190 A, the current density and arc pressure during the EP stage is lower than those recorded during the EN stage. Furthermore, as *I*_ep_ increases, the current density and pressure of the arc during the EP stage progressively approach the values observed in the EN stage. However, once *I*_ep_ exceeds 190 A, both the current density and pressure of the arc during the EP stage surpass those in the EN stage.

**Figure 11:**
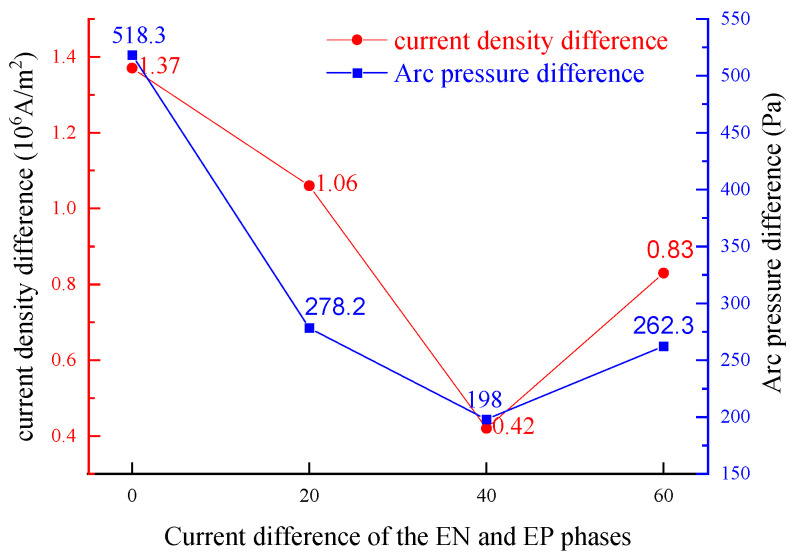
Impact of increasing current during EP phase on the output characteristics of VPPA.

As illustrated in Figure 11, the difference in arc current density (Δ*C*_d_) and arc pressure (Δ*P*) at the central axis on the workpiece surface between the EN and EP stages first decreases and then increases as the current difference (Δ*I*) increases. Specifically, when *I*_en_ is 150 A and *I*_ep_ increases from 150 A to 190 A, Δ*C*_d_ decreases from 1.37 × 10^6^ A/m^2^ to 4.6 × 10^5^ A/m^2^, and Δ*P* decreases from 518.3 Pa to 198 Pa. Both Δ*C*_d_ and Δ*P* increase as *I*_ep_ continues to increase. Furthermore, when the *I*_ep_ is set at 190 A (with Δ*I* being 40 A), the disparity in the thermal-force properties of the arc between the EN and EP stages is minimized, and the corresponding current difference is defined as the “critical current difference”. The fluctuation in arc current density and arc pressure between the EN and EP stages, particularly near the central axis, significantly affects the stable penetration of the keyhole, which in turn influences the consistent weld formation. The above analysis indicates that when the Δ*I* between the EN and EP phases reaches the “critical current difference”, the thermal and mechanical conditions of the EN and EP arcs near the central axis of the workpiece become increasingly consistent. Consequently, the thermal-mechanical outputs of the corresponding EN and EP arcs demonstrated enhanced stability, promoting the stable formation of the weld pool and ultimately leading to improved weld quality.

### 3.2. Influence Mechanism of Welding Current on Keyhole Behavior

**Figure 12:**
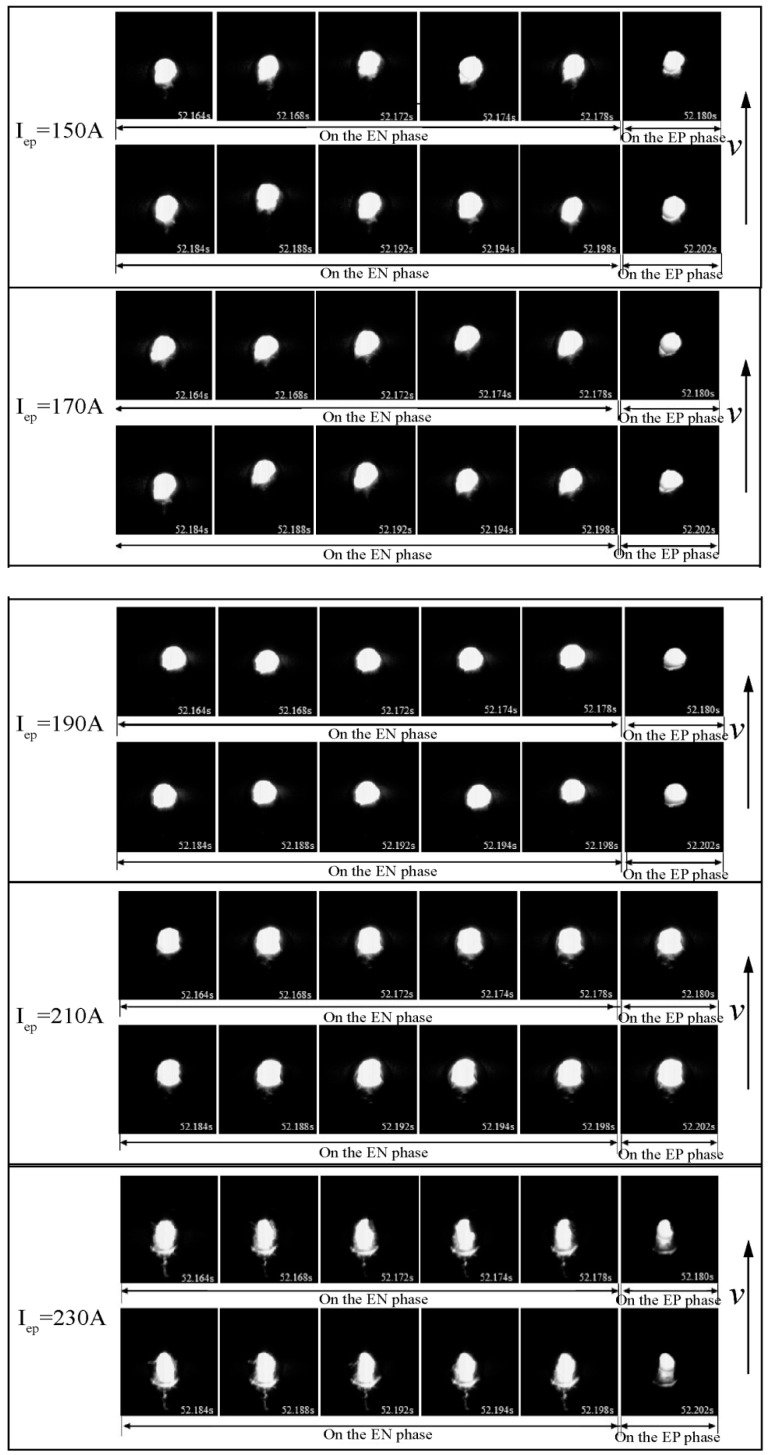
Time-dependent variations in the keyhole with varying current conditions.

The original image of the keyhole on the backside of the weld was collected using an image acquisition system, with *I*_en_ at 150 A and *I*_ep_ at 150 A, 170 A, 190 A, 210 A, and 230 A, respectively, and *v* was the welding speed. Figure 12 illustrates the temporal variation in keyhole dimensions under varying current conditions. When *I*_ep_ is either below or above 190 A, the dimensions of the keyhole fluctuate dramatically, resulting in poor stability. Conversely, at *I*_ep_ = 190 A, the keyhole dimensions remain relatively constant, demonstrating markedly improved stability.

**Figure 13:**
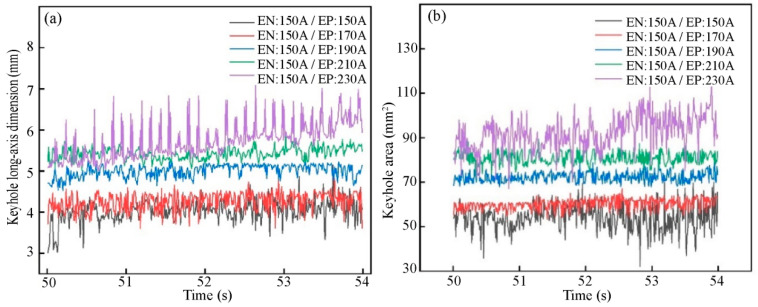
The dimension variations of the keyhole with different currents: (**a**) keyhole long-axis dimension; (**b**) keyhole area.

To quantify the impact of welding current on the characteristic size of the keyhole, edge image extraction was performed on the keyhole image. Subsequently, the keyhole boundary was nonlinearly fitted using the least squares method to obtain the characteristic size of the keyhole. As shown in Figure 13, the long-axis keyhole size refers to its size in the direction of welding, extending through the center of the keyhole, whereas the keyhole area represents the elliptical surface area of the keyhole region. When *I*_en_ is set at 150 A and *I*_ep_ at 190 A (the current difference is 40 A, referred to as the “critical current difference”), the keyhole long-axis dimension and keyhole area have a modest fluctuation range (4.5–5.2 mm and 78–83 mm^2^, respectively), indicating stable keyhole formation. When *I*_ep_ deviates from 190 A, keyhole stability decreases, adversely affecting weld formation. Additionally, the weld formation results illustrated in Figure 2 demonstrate that the optimal weld formation occurs at a current of 190 A, underscoring the importance of maintaining keyhole characteristic dimensions within a specific range for high-quality weld formation.

## 4. Conclusions

A three-dimensional magnetohydrodynamics arc model with alternating loading of the EN and EP polarity arcs was developed to quantitatively analyze the arc characteristics. Additionally, this study elucidated the quantitative influence of welding current on keyhole behavior. The main findings from this research are summarized as follows.

1. When *I*_en_ and *I*_ep_ are equal, the arc pressure and plasma flow velocity at the center of the workpiece during the EN stage exceed those in the EP stage, resulting in increased arc penetration and keyhole digging in the EN stage arc. Although the peak values of arc current density and temperature in the EP stage are lower than those in the EN stage, the arc distribution range in the EP stage is more widespread, leading to greater arc divergence.
2. The heat and force of an arc exhibit periodic fluctuations related to its polarity. The arc thermal-force fluctuations between the EN and EP stages corresponding to the “critical current difference” are minimized, promoting the stable output of the arc heat and force.
3. The fluctuation in the long-axis keyhole size and keyhole area corresponding to the “critical current difference” is minimal, with the keyhole long-axis size fluctuating within 4.5–5.2 mm and the keyhole area within 78–83 mm^2^. However, when the current difference deviates from the “critical current difference”, both the long-axis size and area of the keyhole exhibit significantly increased fluctuations. This instability can lead to an unstable keyhole molten pool and subsequently result in suboptimal weld formation.

## Figures and Tables

**Table 1:** Chemical composition of 2A14 aluminum alloy and ER2319 expressed as weight percentage (%) [19,20].

Material	Cu	Mn	Fe	Si	Ti	Mg	Zn	Al
2A14 aluminum alloy	4.30	0.62	0.56	1.06	0.12	0.65	0.24	Balance
ER2319	6.14	0.24	0.08	0.83	0.14	0.01	0.07	Balance

**Table 2:** The welding parameters during the welding procedure.

Case	*I*_en_	*I*_ep_	*Q*
a	150	150	2.5
b	150	170	2.5
c	150	190	2.5
d	150	210	2.5
e	150	230	2.5

## Data Availability

The original contributions presented in this study are included in the article. Further inquiries can be directed to the corresponding authors.
